# Challenging the “Inoffensiveness” of Regular Cannabis Use by Its Associations with Other Current Risky Substance Use—A Census of 20-Year-Old Swiss Men

**DOI:** 10.3390/ijerph7010046

**Published:** 2010-01-04

**Authors:** Gerhard Gmel, Jacques Gaume, Carole Willi, Pierre-André Michaud, Jacques Cornuz, Jean-Bernard Daeppen

**Affiliations:** 1 Alcohol Treatment Centre, Lausanne University Hospital CHUV, Mont-Paisible 16, 1011 Lausanne, Switzerland; E-Mails: Jacques.Gaume@chuv.ch (J.G.); Jean-Bernard.Daeppen@chuv.ch (J.-B.D.); 2 Swiss Institute for the Prevention of Alcohol and Drug Problems, Case postale 870, 1001 Lausanne, Switzerland; 3 Centre for Addiction and Mental Health, 250 College St, Toronto, Ontario, M5T 1R8, Canada; 4 University of the West of England, Frenchay Campus Coldharbour Lane, Bristol BS16 1QY, UK; 5 Institute of Social and Preventive Medicine, University of Lausanne, Rue du Bugnon 44, 1011 Lausanne, Switzerland; E-Mails: Carole.Willi@chuv.ch (C.W.); Jacques.Cornuz@chuv.ch (J.C.); 6 Research Group on Adolescent Health, Institute of Social and Preventive Medicine, University of Lausanne, Av. de Beaumont 48, 1012 Lausanne, Switzerland; E-Mail: Pierre-Andre.Michaud@chuv.ch; 7 Department of Ambulatory Care and Community Medicine, University of Lausanne, Rue du Bugnon 44, 1011 Lausanne, Switzerland

**Keywords:** risky cannabis use, co-occurring risky licit substance use, early onset, Switzerland

## Abstract

3,537 men enrolling in 2007 for mandatory army recruitment procedures were assessed for the co-occurrence of risky licit substance use among risky cannabis users. Risky cannabis use was defined as at least twice weekly; risky alcohol use as 6+ drinks more than once/monthly, or more than 20 drinks per week; and risky tobacco use as daily smoking. Ninety-five percent of all risky cannabis users reported other risky use. They began using cannabis earlier than did non-risky users, but age of onset was unrelated to other risky substance use. A pressing public health issue among cannabis users stems from risky licit substance use warranting preventive efforts within this age group.

## 1. Introduction

Experts have repeatedly seen cannabis use as being relatively inoffensive, harmless or at least no more dangerous than alcohol and tobacco use [1–5]. There is an ongoing debate about the potential of cannabis use to induce psychotic or affective mental disorders [6,7] (see also related comments), and the detrimental effects of cannabis on pulmonary function is known (e.g., [8]). Risky cannabis use has been largely discussed for its potential as a gateway drug leading to the use of more destructive illicit drugs [9]. The present study on a census of 20-year-old Swiss men adds an additional perspective by hypothesizing that risky cannabis use is no less dangerous than alcohol and tobacco use, since risky cannabis users are often risky tobacco or risky alcohol users as well.

Multiple substance use commonly begins with alcohol or tobacco use, then progresses in some individuals to heavy drinking and marijuana use, followed by other hard drugs [9]. Two major theories describe the etiological sequence. A causal, progressive pathway is suggested by the gateway model, whereas the common liability model posits a mechanism of shared genes and shared environment that accounts for cannabis and other licit and illicit drug use together [10,11]. This latter theory involves the existence of a ‘general syndrome of deviance’ [12] or a ‘problem behaviour syndrome’ [13], which may be related to general behavioural disinhibition [14,15] and related personality traits such as novelty-seeking [16,17], often appearing as a marker for early onset substance use and abuse. Regular cannabis use is associated with early onset that co-occurs with heavy licit and (consequently) hard drug usage [18–20]. In our opinion, the whole discussion of gateway versus common liability mechanisms fails to acknowledge that these two theories are not necessarily contradictory [18,21–23]. The existence of poly-substance use among adolescent and young adult cannabis users needs consideration independent of whether any progression to harder drugs is evident.

Studies of adolescents and young adults worldwide have shown that cannabis, alcohol and tobacco use strongly overlap, particularly among early-onset cannabis users. For example, Kokkevi *et al.* [24] showed that early cannabis use is associated with frequent alcohol and tobacco use across different European countries. Most studies in young populations examine relatively low-risk substance use, even when it involves multiple drugs. In a region like French-speaking Switzerland, over 90% of young men use alcohol, thus it is not surprising that nearly all cannabis users also drink. The present study expands on this by looking at co-occurring patterns of risky cannabis, tobacco and alcohol use.

Several studies extend beyond employing simple definitions of use. Ogborne and Smart [25] found that frequent cannabis use was associated with heavy drinking and driving under the influence. Using biological markers, Kapusta *et al.* [26] showed that high levels of cannabis use were associated with high levels of nicotine dependence in 18-year-old men, which in turn was associated with high levels of alcohol abuse and dependence. It has been shown that about half of all daily smokers meet criteria for tobacco dependence [31,32], although this sometimes happens before the onset of daily smoking [33]. Most correlates of tobacco dependence are also found for daily smoking [34]. Definitions of “risky use” present some difficulties. For example, the European Monitoring Centre for Drugs and Drug Addiction (http://www.emcdda.europa.eu/html.cfm/index31435EN.html; accessed on 14 December 2009) currently has no agreed-upon definition of problematic, risky or dependent cannabis use in general population surveys. Graham and Maslin [27] defined problematic cannabis use as “persistent or recurrent social, occupational, psychological or physical problems related to use, or recurrent dangerous use, or dependence”. Thomas and colleagues [28] developed a typology of cannabis users incorporating quantity, frequency, intensity (e.g., spread over different years) and context of use (e.g., before work or school or alone). In their research, using at least once per week was classified as moderate or even high risk/dependent use. These definitions make it difficult to assess cannabis risk when using screening questionnaires that are often limited to several items that attempt to cover other substances as well. Operational definitions of “risky” or “problematic use” range from once in the past 12 months [29] to over 3 or more times in the past month [30] to at least weekly [25]. In the present study, we used the definitions of “more often than once per week” for risky cannabis use and “daily smoking” for risky tobacco use. Operational definitions for risky alcohol use are even more complex since risk is related to both volume of drinking and heavy alcohol use on single occasions (see below in *2.2. Measures*).

In addition to risky licit substance use and risky cannabis use occurring together, the notion of “reverse gateway” exists [35]. Several studies suggest a progression from cannabis use to tobacco dependence, or the reinforcing effects of cannabis use on tobacco use [35–37]. Heavy smoking or dependence is related to heavy or dependent alcohol use [38,39]. There is also increasing evidence of an association between persistent cannabis use and alcohol dependence [40,41]. The existence of repeated cannabis use is relatively stable from adolescence to adulthood [41], and for Australia, New Zealand and the USA it has been shown that about one out of six or seven ever-users of cannabis become dependent on it [42]. The transition from cannabis use to dependence is reportedly more common among individuals with earlier onset and concomitant use of other legal and illegal substances [43,44]. Risky licit substance use related to risky cannabis use is a major public health issue. Alcohol and tobacco use are among the leading risk factors for mortality and morbidity worldwide and within developed countries [45], and alcohol use is the most important risk factor among adolescents and young adults [46]. Patton *et al.* [35] argue that if the link between risky cannabis use and risky other substance use is causal, then the increase in risky use of licit substances like tobacco would be the most important health consequence of cannabis use. Although the present study is cross-sectional in nature and cannot address causal paths, it does attempt to highlight the association of risky cannabis use with risky licit drug use (particularly when it occurs early).

## 2. Methods

### 2.1. Sample

Sampling took place each week between January 23 and August 29, 2007 in the recruitment centre at Lausanne, except for holiday closures during six of these weeks. Switzerland has a mandatory two-day army recruitment process and virtually all males complete the physical, medical and cognitive assessments for service eligibility in the army by age 20. The Lausanne centre processes all men in the French-speaking sector, which comprises about 21% of the Swiss population [47]. Those with documented severe disablement are excused from this procedure and according to estimates by the army, number less than 3%. Women may voluntarily join the army, but in the present study, only eight showed up to participate. They were not included.

From the total of 4,116 men who showed up during the roughly 25 weeks, 264 were never seen by the research staff because of early discharge from the army for mental and physical handicaps that *a priori* precluded any service or completion of the assessment process. The remaining 3,852 conscripts were invited to fill out a 5-minute screener for alcohol, tobacco and illicit drug use. The present study is part of a larger project providing brief interventions to conscripts with a six-month follow-up, but only screening data are used herein. All subjects were informed that participation in the study was voluntary and that any data provided would never be turned over to nor seen by anyone in the army. Only 289 men refused the screening and another 24 could not finish the questionnaire because they were called out to complete other mandatory army tasks. Two more were excluded due to apparent inconsistent or falsified answers (e.g., one non-drinker claimed to have had more than 100 drinks the week before the interview, and another individual reported daily intake of more than 100 drinks). The end sample included analysable data from 3,537 young men. The Ethics Committee for Clinical Research at the Lausanne University Medical School approved this study.

### 2.2. Measures

The screening questionnaire assessed tobacco, drug and alcohol consumption in the past six months. A general reference period of six months was chosen to exclude overlap of substance use behaviours for the brief intervention study and follow-up six months later. Cannabis items referred to frequency of use both in the past six months and in the past 30 days. Risky cannabis use was defined as twice a week or more during the past six months, or more than once per week during the past 30 days. We generally addressed the past six months because this was the wider reference period. However, if use in the past 30-day was more frequent, this measure was used as it typically contains less recall bias.

Tobacco use questions differentiated between regular and occasional smoking in the past 6 months and (among current smokers) assessed daily smoking and number of cigarettes per day. Risky tobacco use was defined as daily smoking.

Usual drinking frequency was assessed with an open-ended question about the average number of days per week on which alcohol was consumed over the past six months. Non-weekly drinkers were given a closed-ended question and selected categories of “2 to 4 times a month” (coded as 42 days per year), “once a month or less often” (coded as nine days per year) and “never”. Usual quantity was assessed with an open-ended question about number of standard drinks per drinking day. Pictures of standard vessels that contain about 10 grams of pure ethanol were shown with the following labels identifying container sizes: 100 mL glass of wine; 250 mL glass of beer; 275 mL bottle of Alcopops (a premixed drink containing spirits); 25 mL glass of spirits; and 50 mL tall glass containing spirits and aperitif (e.g., martini). The number of drinks per drinking day was multiplied by number of drinking days to obtain the weekly drinking volume. Conscripts were also asked retrospectively to itemize in a one-week diary their daily beverage-specific consumption, using the alcohol definitions listed above. Drinks were summed over beverages for each day and totalled over seven days. Risky volume drinking was defined as 21 drinks per week on average on either of the two measures. This represents a compromise between the 14 drinks cut-off for brief interventions recommended by the National Institute on Alcohol Abuse and Alcoholism [48], and the 28 drinks cut-off for harmful use working definition of the World Health Organization [49].

The frequency of risky single occasion drinking (RSOD) was measured with an open-ended question about usual number of days per month on which 6+ drinks were consumed. Six drinks contain approximately 60 grams of pure alcohol and equal the most common US measure of 5+ drinks of 12 grams per drink [50]. Risky alcohol use was defined as either risky volume drinking or RSOD at least twice per month (congruent with a widely used cut-off for RSOD in the United States [50]).

Ages of onset for cannabis use, smoking and drunkenness were also measured. Unfortunately, age of onset for alcohol use or daily smoking was not available from the short screening instrument.

### 2.3. Statistical Analysis

Statistics such as ANOVA F-tests for comparisons of means and *X*^2^ tests for comparing prevalence rates were utilized. In addition, three multiple logistic regressions were performed, where each risky use was the dependent variable and the risky use of the remaining two substances (and its interactions) were the independent variables. Interactions were evaluated through graphic displays.

## 3. Results

The mean age of the subjects was 19.94 (SE = 0.025); 0.6 % were younger than 18, and 14.8% were older than 20. Table 1 shows that nearly all subjects (92.8%) consumed alcohol and that 58.1% of these drank in a risky way (*i.e.,* had at least two RSOD occasions monthly, or averaged more than 20 drinks per week). Almost 37% smoked daily and more than 17% were risky cannabis users (*i.e.,* at least twice weekly). Given that alcohol use is so widespread, it was not surprising that the exclusive use of cannabis or tobacco was rare. Only 0.4% of the sample used cannabis only, and among all risky cannabis users, only 4.9% identified it as their sole risky use. Only 5.5% of all men reported not using any of the three substances in the past 6 months.

Table 2 shows high prevalence rates for risky use of legal substances, with co-occurring risky use most prevalent for tobacco and alcohol (16.2%). The second most prevalent combination was self-reported risky use of tobacco, alcohol and cannabis (10.6%). Tobacco use commonly had an earlier age of onset than did cannabis and drunkenness.

As shown in Table 3, risky alcohol use can occur alone (about 48% of risky alcohol users did not use any other risky substance), but this was rarely the case for risky tobacco and cannabis use. Nearly two-thirds (62%) of risky cannabis users reported both risky alcohol and tobacco use as well, and nearly all (95%) of them used at least one other substance concomitantly in a risky way.

**Figure 1.**
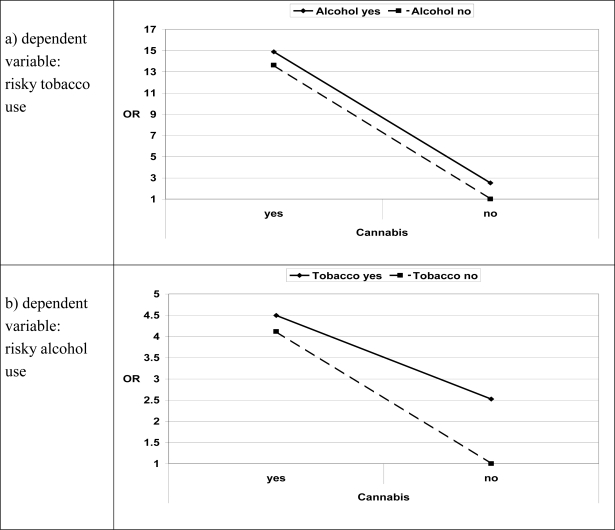

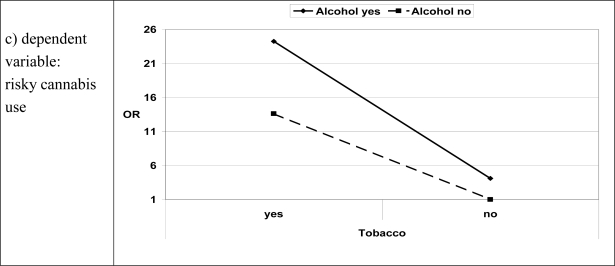
Interplay of risky substance use, odds ratios* for alternating dependent and independent variables. Odds ratios from logistic regressions, 95% confidence intervals in parenthesis; all coefficients were significant at p <0.001 except for the constant of model b) with p = 0.016: Model a) risky cannabis use: 13.62 (8.83–21.01), risky alcohol use: 2.52 (2.13–2.99), interaction: 0.43 (0.26–0.71), constant: 0.23 (0.20–0.26). Model b): risky cannabis use: 4.11 (2.72–6.21), risky tobacco use: 2.52 (2.13–2.99), interaction: 0.43 (0.26–0.71), constant: 0.90 (0.83–0.98). Model c) risky tobacco use: 13.62 (8.83–21.01), risky alcohol use: 4.11 (2.72–6.21), interaction: 0.43 (0.26–0.71), constant: 0.03 (0.02–0.04).

Odds ratios for alternating risky substance use variables as dependent or independent variables (including the interactions of the independent variables) were calculated and graphically presented in Figure 1 in order to make the interactions easier to interpret. Risky cannabis use showed an important association with risky tobacco use (and vice versa), whereas risky alcohol use added relatively little to the cannabis effect. For example, among non-risky alcohol users the odds ratios for risky tobacco use (daily smoking) increased from 1 to more than 13 with risky cannabis use. The odds ratios for risky alcohol use increased the odds ratios additionally by less than 2 (see panel a) in Figure 1). Risky cannabis use was more strongly associated with risky alcohol use (e.g., an increase in odds ratios from 1 to 4.11 for risky alcohol use when there was risky cannabis use but no risky tobacco use, see panel b) in figure 1) than with risky tobacco use. The combination of both added relatively little to the effect of risky cannabis use alone (risky tobacco use increased the odds ratios from 4.11 to 4.49 for risky alcohol use among risky cannabis users, see panel b) in Figure 1). Both risky tobacco use (odds ratio = 13.62 among non-risky alcohol users, see panel c) in Figure 1), and risky alcohol use in combination with risky tobacco use (odds ratio = 24.26, see panel c) in Figure 1), increased the odds for risky cannabis use.

Table 4 shows that former cannabis users reported a significantly later age of onset than did present users. These age differences, however, were more pronounced among risky users, who started using cannabis more than a year earlier than did non-risky users. Interestingly, among risky cannabis users no significant differences in other risky substance use were found between those with cannabis use onset ages earlier than 16 compared to those with onset ages 16 and older.

## 4. Discussion

Cannabis use is sometimes seen as a relatively inoffensive or harmless substance compared to legal substances such as tobacco and alcohol, but risky users appear to be more prone to engaging in other risky behaviours. The present study reveals that in the past six months, ninety-five percent of the subjects used either tobacco or alcohol in a risky way, and nearly two-thirds of them did both. Tobacco and alcohol use have been shown to be two of the three main risk factors for mortality and morbidity in developed societies [45]. This is a major public health concern, since 17.2% of all young Swiss francophone men use cannabis in a risky way (*i.e.,* at least twice per week).

The present study is cross-sectional in nature, therefore it cannot show whether cannabis is indeed a causal risk factor for legal substance use. In addition, the average progression from initiation of smoking to drinking (drunkenness) to cannabis use is contrary to the notion that cannabis use causes later legal substance use. As Hall [51] suggests, longitudinal studies of the developmental effects of cannabis use in adolescence on outcomes in adulthood would be needed in order to assess whether adverse health effects are causally related to cannabis use. A longitudinal approach would still be problematic, because young cannabis users differ from their peers in various ways (e.g., other substance use, parental characteristics, socioeconomic background, academic performance, and antisocial traits). It would be difficult to disentangle the effects of cannabis from those due to a common liability [7]. Similarly, in conjunction with the gateway hypothesis (*i.e.,* the progression from legal substance use to regular cannabis use to harder drug use), it has been argued that selective recruitment into early cannabis use by socially deviant young people may explain why some individuals progress to other risky substance use behaviours [51]. The observed sequence could be explained by the easy availability of different drugs, along with a pre-existing propensity to use any type of drug.

The motivation for the present study is not to provide further evidence for or against the gateway theory. There is increasing evidence that cannabis use that is more intense than just casual or occasional use may reinforce smoking and increase the risk for tobacco dependence [35–37]. It may also increase the risk for alcohol dependence, directly [40,41] or indirectly through tobacco dependence [38,39]. The finding that the age of onset for cannabis use was independent of other risky substance use is indicative of a reverse causation. As predicted by a common liability model, there were twice as many risky cannabis users with onset before age 16. However, once cannabis is used in a risky way the differences in the proportions of other risky licit substance use by those with earlier and later onset is minimal. This is contrary to what would be expected if common liability is the main reason for the co-occurrence of risky substance use. Given the high cannabis use prevalence in Switzerland (see also next paragraph) and the strong link with other risky use, the public health effect seems to stem from the impact of a large part of the general population at this age, not from a small group with a high common liability.

Controversy surrounding the gateway versus the common liability paradigms certainly has implications for designing future preventive actions. Should cannabis be taken from the black market (which provides a shared environment with harder drugs) and should prevention efforts focus on early childhood development, or must the sequence of drug use be interrupted in order to eliminate a “causal progression” to hard drugs [11,52]? We believe that interventions on early childhood development within high-risk groups are always useful. Generally, very few individuals who use cannabis progress to harder drugs, therefore the potential of this substance to act as a gateway drug is probably of minor public health relevance. We agree with Patton *et al.* [35] that one of the major public health concerns about the use of cannabis is its high rate of co-occurrence with risky tobacco and alcohol use. It is a particular public health concern for Switzerland, which is one of the leading countries in adolescent cannabis use [53,54], so it seems unlikely that risky cannabis use is a problem limited only to a small high-risk group with a common liability for substance use disorders.

A major limitation of the present study is that it applies only to Francophone young men. German-speaking men will be studied in research scheduled to begin in 2010. The previously mentioned Youth studies [53,54], however, did show that there are few differences across linguistic regions and that cannabis use is very high among Swiss girls too.

In conclusion, the interesting debate over common liability and gateway hypotheses and the implications for future primary preventive efforts seems to overshadow the urgent need for current secondary prevention. From our point of view, for secondary prevention it does not matter whether a common liability or a causal substance use sequence leads young men to cannabis use. Young risky cannabis users (at least in the French-speaking parts of Switzerland) are very likely to suffer from some health consequences related to their concomitant risky use of tobacco and alcohol. We believe that (independent of whether cannabis itself creates serious health consequences) frequent use of this substance is a strong marker for likely adverse health consequences, arising from the use of licit substances. To counteract this, a good starting point for preventive actions would be brief motivational interviewing interventions, which should not only target cannabis as the primary substance, but should take into account other multiple risky substance use behaviours among young men.

## Figures and Tables

**Table 1.** Six months prevalence rates of alcohol, tobacco and cannabis use and of risky use of each substance (n = 3537).

	**Use (100% = all men)**	Exclusive use of substance*	**Risky use (100%=all men)**	Exclusive risky use of substance**
Tobacco	51.2%	1.7%	36.6%	19.5%
Alcohol	92.8%	38.2%	58.1%	48.5%
Cannabis	38.2%	0.4%	17.2%	4.9%

Risky use: Alcohol: RSOD at least twice per month or usual consumption of more than 20 drinks a week. Tobacco: daily smoking. Cannabis: cannabis use at least twice weekly.

* 100% = all past six months users.

** 100% = all risky users of the substance.

**Table 2.** Prevalence (in %) of co-occurrence of risky use (alcohol, tobacco, and cannabis) in the past six months, and age of onset of behaviors*.

**Risk groups**	**n**	**%**	**Behaviour onset in years among risky users (SD)**		
			**Tobacco**	**Drunkenness**	**Cannabis**
No risky use	*1107*	31.3			
Tobacco	*252*	7.1	14.7 (2.19)		
Alcohol	*996*	28.2		15.1 (1.54)	
Cannabis	*30*	0.8			14.9 (1.91)
Tob & alc.	*572*	16.2	14.3 (2.12)	14.6 (1.60)	
Tob. & can.	*93*	2.6	13.1 (2.42)		14.5 (2.06)
Alc. & can.	*111*	3.1		14.3 (1.84)	14.9 (1.67)
Tob. & alc. & can	*376*	10.6	13.6 (2.05)	14.0 (1.61)	14.6 (1.89)

* age of onset was measured as first cigarette use, first time drunkenness, and first cannabis use.

Risky use: Alcohol: RSOD at least twice per month or usual consumption of more than 20 drinks per week. Tobacco: daily smoking. Cannabis: use at least twice weekly.

**Table 3.** Co-occurrence of risky use (n = 3537) in the past six months.

	**Risky tobacco**	**Risky alcohol**	**Risky cannabis**	**At least one of the other two**	**Both**
Risky tobacco use	-	73%	36%	81%	29%
Risky alcohol use	46%	-	24%	52%	18%
Risky cannabis use	77%	80%	-	95%	62%

Risky use: Alcohol: RSOD at least twice per month or usual consumption of more than 20 drinks per week. Tobacco: daily smoking. Cannabis: use at least twice weekly.

**Table 4.** Mean ages of cannabis use onset for ex- and current past six months cannabis users, risky and non-risky past six months users, and prevalence of risky alcohol and tobacco use by early versus late cannabis use onset.

	**Cannabis use status**				**Statistic (p-value)**
	Ex-user (n = 773)	Past six months use (n = 1,351)			
		
Mean age of onset	15.60	15.25			18.62 (<0.001)*
			
		Non-risky use (n = 741)	risky use (n = 610)		
			
Mean age of onset		15.74	14.66		122.4 (<0.001)*
				
Other risky behaviours:			Onset <16 years (n = 409)	Onset 16+ years (n = 201)	
				
- % no other risk			4.4	6.0	3.13 (0.372)**
- % risky tobacco use			15.4	14.9	
- % risky alcohol use			16.6	21.4	
- % risky tobacco and alcohol use			63.6	57.7	

* Tests for age of onset: ANOVA;

** Test for risky substance use: Overall *X*^2^ for the 2*4 table.
